# SUPPORTING DEMENTIA CAREGIVERS: THE PUBLIC HEALTH CENTER OF EXCELLENCE ON DEMENTIA CAREGIVING

**DOI:** 10.1093/geroni/igac059.275

**Published:** 2022-12-20

**Authors:** Joseph Gaugler

**Affiliations:** University of Minnesota, Minneapolis, Minnesota, United States

## Abstract

The Public Health Center of Excellence on Dementia Caregiving has established a robust, national network of 17 organizations to jointly develop and disseminate information, tools, and resources to support families, friends, and other unpaid caregivers of people living with dementia. The Center is establishing partnerships with local, state, tribal, and other public health organizations throughout the United States to provide technical assistance on the sustainability of dementia caregiving support programs as well as the cultural adaptation of messaging and tools to best meet the needs of diverse communities The Center also has a robust stakeholder engagement process so that the voices of caregivers and public health providers are incorporated in all Center efforts. The Center also collaborates with the Public Health Centers of Excellence on Risk Reduction and Early Detection to develop and refine cross-cutting resources and tools to further meet the complex public health challenges of dementia.

